# Epidemiological Profile of Chronic Wounds in an Indian Population: A Community-Based Cross-Sectional Observational Study

**DOI:** 10.7759/cureus.68684

**Published:** 2024-09-05

**Authors:** Aditya Sharma, Vivek Srivastava, Ravi Shankar, Ashish K Yadav, Mumtaz A Ansari, Sanjeev K Gupta

**Affiliations:** 1 General Surgery, Institute of Medical Sciences, Banaras Hindu University, Varanasi, IND; 2 Community Medicine, Institute of Medical Sciences, Banaras Hindu University, Varanasi, IND; 3 Biostatistics, Institute of Medical Sciences, Banaras Hindu University, Varanasi, IND

**Keywords:** chronic disease epidemiology, chronic wounds, community health and primary health care research, healthcare burden, non-healing wounds, prevalence

## Abstract

Introduction

A wound that has not healed in a timely and organized manner to maintain its anatomical and functional integrity is considered chronic. It can last anywhere from four weeks to over three months and could be caused by trauma, diabetes, vascular disease, infection, or pressure. The primary objective of the study was to determine the prevalence of wounds in a rural and urban community in India, and the secondary objective was to evaluate the effect of various chronic wounds on quality of life (QoL) as assessed by the Short-Form Health Survey-36 (SF-36) questionnaire.

Materials and methods

The present study is a cross-sectional analytical study done between June 2022 and December 2023, and a total of 10,003 sample population was screened in Varanasi district, Uttar Pradesh, India. The study involved conducting a door-to-door survey of the population sample under interest based on a stratified sampling technique. During the survey, the epidemiological profile of the sample population, a detailed history, and clinical examination findings were recorded in a proforma. The reports of relevant diagnostic investigations were also recorded.

Results

The overall prevalence of chronic wounds was 1.89 per 1000 (19/10003) population. The prevalence of wounds in urban areas was 1.57 per 1000 (11/6984) population, while that in rural communities was 2.64 per 1000 (8/3019) population. Of the total 19 patients with chronic wounds, the majority of the patients, i.e., seven each (36.8%), had diabetic foot ulcers (DFUs) and neglected/uncared trauma as the etiology for chronic wounds. The remaining five (5.3%) patients were equally divided between cellulitis, venous ulcers, trophic ulcers, tubercular ulcers, and malignancy as the etiology for chronic wounds.

The QoL data suggested that 11 (57.9%) patients reported their general health status as fair with wounds, followed by five (26.3%) reporting their general health status as poor. Only three (15.8%) of the patients with wounds reported their general health condition as good. There was no significant difference between the QoL of rural and urban patients with chronic wounds.

The overall prevalence of wounds has decreased from 15.03 to 1.89 per 1000 people in the survey locality. The switch from opting for home-based remedies to supervised care under the clinician (be it in a private or public setting) clearly indicates raised awareness regarding the management of wounds, and with time, people have shown an increased interest in healthcare facility treatment under supervision over adopting home remedies for wound treatment. The same domain may be attributed to the prompt management and efficacy of the management protocols by the treating physicians in India.

Conclusion

The findings of the present study regarding the incidence and prevalence of chronic wounds in connection to demographic factors are important when allocating resources and making healthcare plans. The present study conveys a clear message to healthcare practitioners about the necessity of carrying out large-scale epidemiological research on this topic and offers a solid foundation for future studies.

## Introduction

The burden of chronic wounds is substantial and frequently underestimated by the patient, the medical community, and society at large [1]. It would be better to call these wounds that fail to heal, not just because of time frame but due to various underlying mechanisms, "complex wounds" [2,3]. According to a preliminary literature review, there is currently no recent data on their prevalence and their exact impact on the quality of life (QoL), especially in the Indian scenario [2,3]. There was a relative lack of current, high-quality data on the number of people suffering from chronic non-healing wounds, the number of resources used by health agencies to manage wounds, and the kind of care provided to those whose wounds are causing them to become unwell [3]. All people experience wounds, and most of them heal without any complications. However, a significant percentage of individuals suffer from wounds that either never heal at all or heal very slowly [4,5]. The focus of the research was on these more serious wounds, which are primarily handled by community nurses and dressers. The most common types of chronic wounds are leg ulcers (mainly from venous and/or arterial disease), pressure ulcers (from unrelieved pressure as a result of immobility), and diabetic foot ulcers (DFUs) (from venous or arterial and neurological diseases) [1-3]. Another area of study that comprises modern epidemiology is the examination of life-related events, facts, and conditions that have an impact on the general population [3]. Similar to public health as an entity, epidemiology usually puts society's overall welfare ahead of an individual's [2,3,4]. It is clear from a research perspective that this kind of comprehension is necessary for determining the need to conduct further research and prioritizing research concerns [3].

A greater understanding is also required for healthcare organizations to ensure that the right facilities are offered to recognize and address staff training requirements and to carry out effective management, preferably at a tertiary healthcare center with a multidisciplinary approach [1,2]. Furthermore, these basic data are needed for value-of-information assessments that will direct future studies or fill cost-effectiveness models currently in use to assess the cost-effectiveness of currently available treatments. It is clear from a research perspective that this kind of understanding is necessary for determining the need to conduct more research and prioritizing research concerns [3,4]. Chronic wounds have long been a major public health concern, but estimates of their exact impact are variable since there are hardly any recent community-based studies, and the data that is available in the literature are hospital-based studies that are easy to conduct but tend to be skewed since such studies do not denote a homogenous population [3]. This information is crucial given the rising frequency of lifestyle diseases because it is necessary to know the incidence and prevalence of chronic wounds in connection to demographic factors when allocating resources and making healthcare plans [4,5]. Keeping this research question in mind, the primary objective of the study was to determine the prevalence of wounds in rural and urban communities of an Indian district. 

## Materials and methods

Study design

The primary objective of the study was to determine the prevalence of wounds in a rural and urban community in Varanasi district, Uttar Pradesh, India, and the secondary objective was to evaluate the impact of various chronic wounds on the QoL as assessed by the Short-Form Health Survey-36 (SF-36) questionnaire (Table 5 and Table 6 of Appendix). The present study is a cross-sectional analytical study done between June 2022 and December 2023. The study was conducted in the Department of General Surgery in collaboration with the Department of Community Medicine and Centre of Biostatistics, Institute of Medical Sciences, Banaras Hindu University, Varanasi, Uttar Pradesh, India. The study was approved by the Institutional Ethics Committee, Institute of Medical Sciences, Banaras Hindu University, Varanasi, Uttar Pradesh, India (ECR/526/Inst/UP/2014/RR-20/dt./19.5.2020/No.Dean/2022/EC/3628). 

Methods

The study involved conducting a door-to-door survey of the population sample under interest based on stratified sampling technique, a probability sampling method that divides a population into homogeneous groups called strata based on shared characteristics and is more economical than other sampling methods as it minimizes research costs by dividing a large population into smaller groups with similar members. Inclusion criteria included subjects born in residential areas of Varanasi District and residing in Sunderpur or Kandwa village for at least 20 years (to exclude the migrant population from other regions and to maintain homogeneity) while the residents who stay temporarily in the said areas and those who refused to give consent were excluded. During the survey of the sample population, the prevalence of chronic wounds was identified and noted. The area under survey was identified in two communities. Sunderpur, a semi-slum area with a population of 27,076 individuals (males: 14,240 and females: 12,836) located 1 km from Banaras Hindu University, Varanasi, was identified as a representation of the urban population of the district. On the other hand, Kandwa village, with a population of 11,685 individuals (male: 6,165 and female: 5,520), located 4 km from Banaras Hindu University, Varanasi, represented the rural population of the district. The wounds were classified as chronic wounds if they had persisted for more than three months [1].

Pilot study (feasibility study)

**Figure 1:**
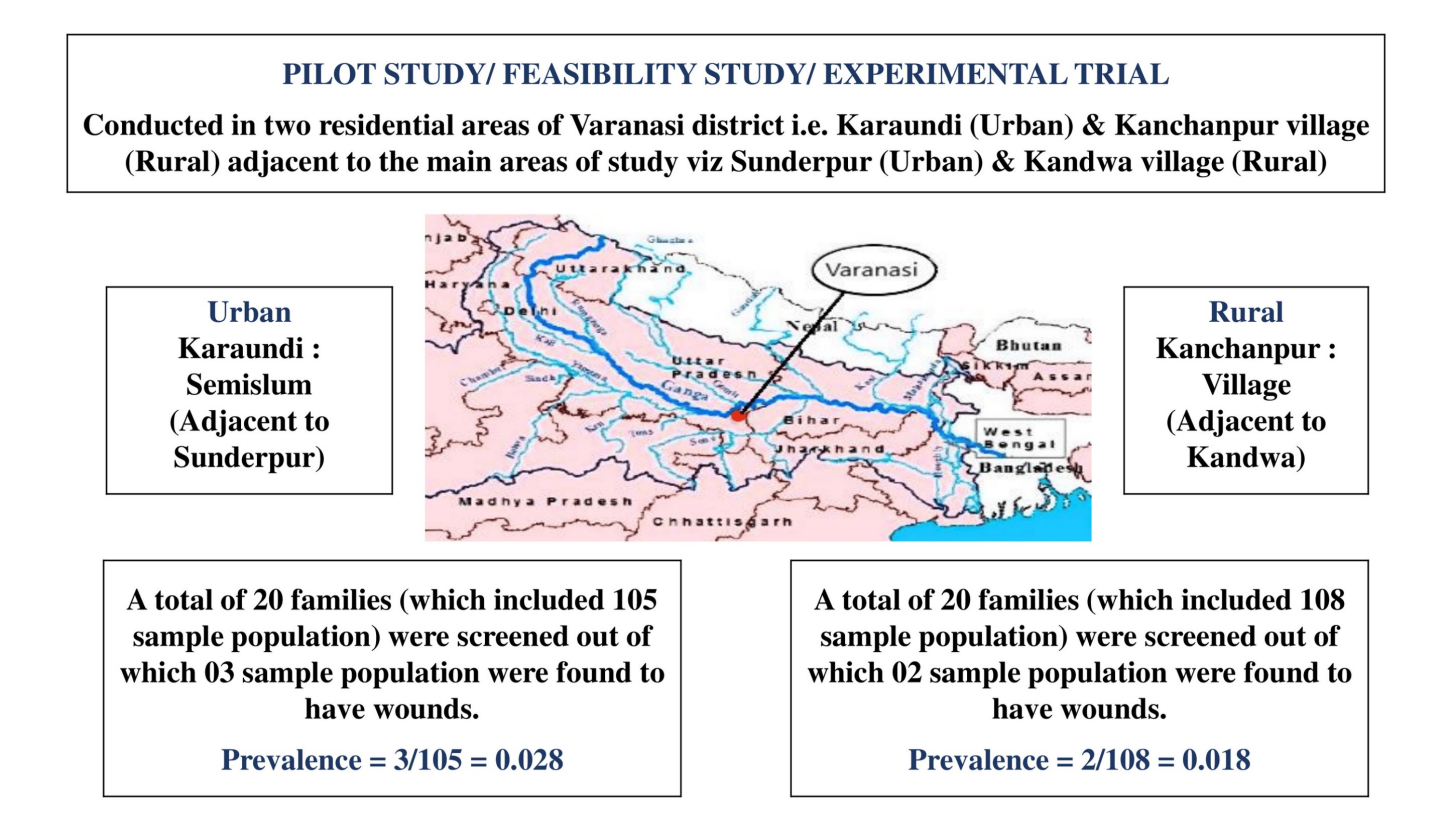
Details of a pilot study conducted in the adjacent areas of the same region in order to determine sample size.

A pilot study or feasibility study was conducted prior to the commencement of the proposed study in order to determine the sample size and the effectiveness of data collection [6]. For this pilot study, the residential areas adjacent to the areas of the main study, i.e., Karaundi (an urban semi-slum adjacent to Sunderpur) and Kanchanpur village (a rural area adjacent to Kandwa village), were undertaken, as shown in Figure 1.

Sample size

**Figure 2:**
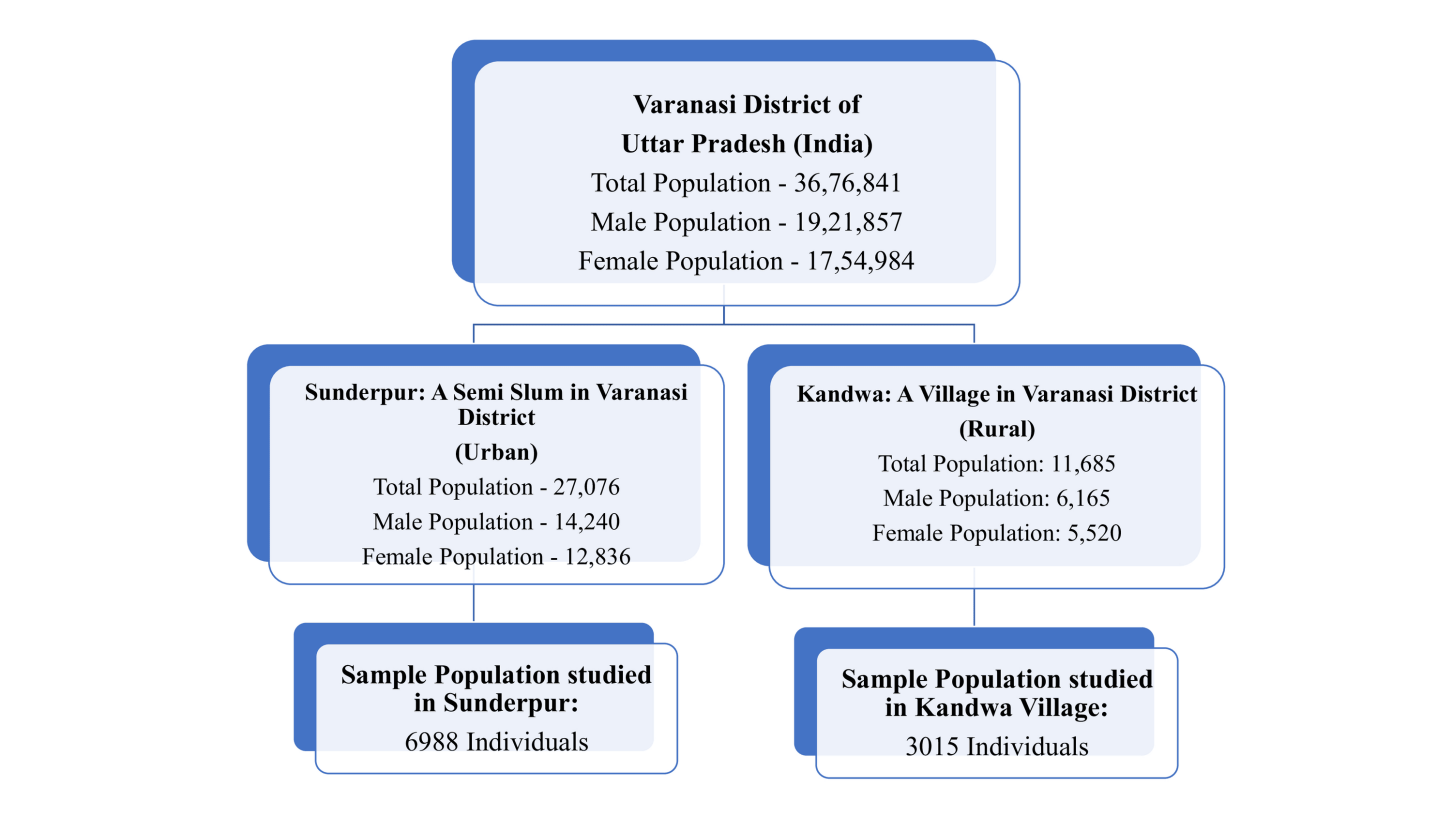
A flow chart showing the sampling done from two study areas as per the PPS sampling method. PPS, probability proportional to size

Based on Yamane's formula and utilizing the prevalence data obtained from the pilot study for both communities, the sample population to be surveyed came out to be 4721 individuals (i.e., the minimum number of subjects required for the above study with 90% power and 95% confidence level). Hence, based on the proportion of population in the Sunderpur and Kandwa areas, the minimum sample to be surveyed was calculated as 3298 for Sunderpur and 1423 for Kandwa. As the survey of the sample population ended well before the study duration, we continued to recruit further population in the same proportion, and the final data collection was 10003 (6988 in Sunderpur and 3015 in Kandwa) individuals for the above study. These samples selected were from two study areas of interest as per PPS (probability proportional to size) sampling. Thus, the final sample sizes from both areas were approximately double the calculated sample size, as shown in Figure 2.

Study tools

A pre-tested, semi-structured questionnaire was used to elicit various types of information from the study participants. The questionnaire had three parts: a brief demographic profile of the family, details of the wound, and the SF-36 questionnaire meant for the assessment of the QoL impacted after having chronic non-healing wounds.

Statistical analysis

The difference in proportion was compared using the Z-test with respect to various socio-demographic variables of patients and the characteristics of various types of wounds, and based on the objectives of the study, descriptive and inferential statistics were drawn. For statistical significance, a p-value of less than 0.05 was considered significant.

## Results

Demographic profile of the community

In this cross-sectional study, 10,003 people in two communities were screened, and a total of 19 patients with chronic wounds were detected. In the present study, the majority (5891, 58%) of the population was in the age range of less than 30 years (p=0.00001). The males (5113, 51.12%) outnumbered the females (p=0.0027). The majority (6719, 67.3%) of the population had attended primary school (p=0.00001). Nearly 6239 (62.37%) of the total population were engaged in outdoor occupations, and 778 (7.78%) individuals were unemployed, including housewives, children, and elderly people (p=0.00001). Only 371 (20.91%) families belonged to the lower class, while 1283 (70.42%) belonged to the lower middle, 149 (8.18%) belonged to the middle, 10 (0.55%) belonged to the upper middle, and 9 (0.49%) belonged to the upper class with respect to the income distribution of the families (p=0.00001). The overall demographic parameters are shown in Table 1.

**Table 1:** The demographic profile of the population in the present study. Sample size (N): 10,003; total families screened: 1822; p-value <0.05 is significant

Demographic variable	June 2022-December 2023		p-value
	Frequency (N)	Percentage (%)	
Age			
Less than 30 Years	5821	58	0.00001
More than 30 Years	4182	41	
Gender			
Female	4890	48.88	0.0027
Male	5113	51.12	
Education			
Below primary school	3284	32.7	0.00001
Above primary school	6719	67.3	
Occupation			
Indoor	2986	29.85	0.00001
Outdoor	6239	62.37	
Income			
Lower class	371	20.36	0.00001
Lower middle/middle/upper middle/upper	1451	79.64	

Socio-demographic profile of patients with wounds

In the present study, only 19 patients, including those with post-traumatic and post-cellulitis wounds (neglected and uncared wounds), were found to have chronic non-healing wounds, while acute wounds were not observed in any of the 10,003 individuals screened, both in urban and rural communities. In urban population, 11 (57.89%) individuals were found to have chronic non-healing wounds of the screened (6984, 69.8%) population, while in rural communities, eight (42.1%) individuals of the screened (3019, 30.2%) population (p=0.49).

The majority of the patients, i.e., 17 (89.47%), who were having wounds fell into the age group of more than 30 years (p=0.131). The patients with chronic non-healing wounds showed a male preponderance, with 13 (68.42%) of males in the total affected population (p=0.131). Nearly 11 (57.89%) of the patients with wounds were engaged in outdoor occupations (p=0.25). The majority of the patients with wounds, i.e., 11 (57.89%), had attended the above primary school. The socio-economic status of the majority of patients having wounds, i.e., eight (42.11%), belonged to the lower middle class, followed by the lower class and upper middle classes, which covered four (21.05%) of the patients, as shown in Table 2.

**Table 2:** The demography of the patients with chronic non-healing wounds. p-value <0.05 is significant. *No observation

Demographic variable	June 2022-December 2023		p-value
Type of wound	Frequency (N)	Percentage (%)	
Acute	*	*	
Chronic	19	100.0	-
Total	19	100.0	
Community	Frequency (N)	Percentage (%)	
Rural	8	42.1	0.49
Urban	11	57.9	
Age (years)	Frequency (N)	Percentage (%)	
Less than 30 years	2	10.53	0.014
More than 30 years	17	89.47	
Gender	Frequency (N)	Percentage (%)	
Male	13	68.42	0.131
Female	6	31.58	
Occupation	Frequency (N)	Percentage (%)	
Outdoor	11	57.89	0.25
Indoor	5	26.32	
Education	Frequency (N)	Percentage (%)	
Below primary school	8	42	0.49
Above primary school	11	58	
Socioeconomic status	Frequency (N)	Percentage (%)	
Lower	4	21.05	
Lower middle	8	42.11	
Middle	3	15.79	-
Upper middle	4	21.05	
Upper	*	*	
Total	19	100.0	

Characteristics of chronic wounds

In the total number of patients with wounds, the mode of onset was trauma in three (15.8%) of the patients, while in 16, (84.2%) it was spontaneous (p=0.0142). In the majority of patients with wounds, i.e., 17 (89.4%), the most common anatomical location was the lower extremity, followed by other locations like the head and neck body and trunk regions, which constituted one (5.3%) each of the affected patients (p=0.0065). The majority (7, 36.8%) had uncared DFUs and neglected/uncared repeated trauma as the etiology for chronic wounds. The remaining (5, 26.31%) patients were equally divided between cellulitis, venous ulcers, trophic ulcers, tubercular ulcers, and malignancy, one (5.3%) each as the etiology for chronic wounds. In the individuals having wounds, most of the patients, i.e., 14 (73.68%), had chronic non-healing wounds for a duration of six months (p=0.06). Most of the patients, i.e., 18 (94.7%), with chronic non-healing wounds complained of associated pain, which was mild in intensity (p=0.003). Only five (26.3) patients with chronic non-healing wounds had associated discharge, which was purulent, noted during the examination of the wounds (p=0.06). The majority, i.e., 12 (63.1%), of the patients with wounds were non-smokers (p=0.27), non-alcoholics, i.e., 13 (68.4%) (p=0.13), and non-vegetarians, i.e., 11 (57.9%) (p=0.49). The majority of the patients, i.e., 13 (68.4%), received primary treatment for wounds from the public sector (p=0.10). Nearly 16 (84.2%) of the patients with chronic non-healing wounds took local and systemic treatment. 

**Figure 3:**
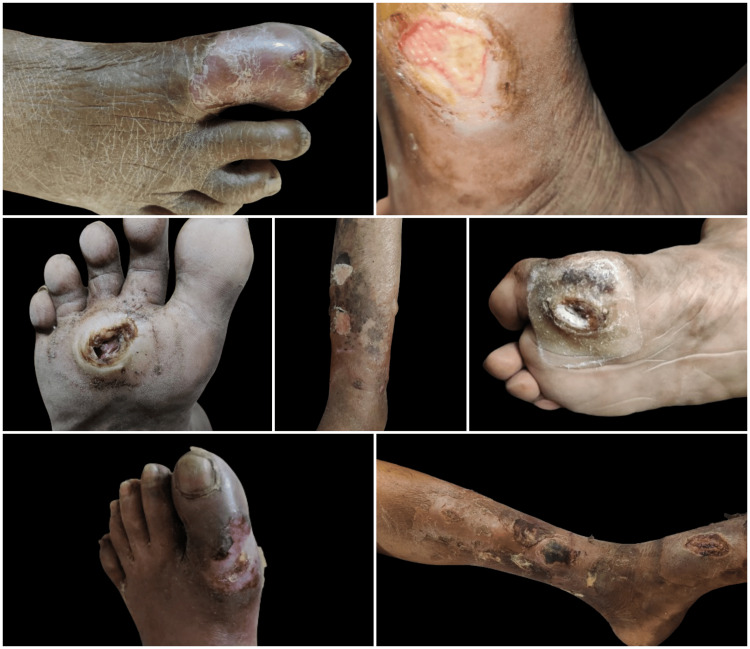
The patients with DFUs found in the present community-based study. DFUs, diabetic foot ulcers

**Figure 4:**
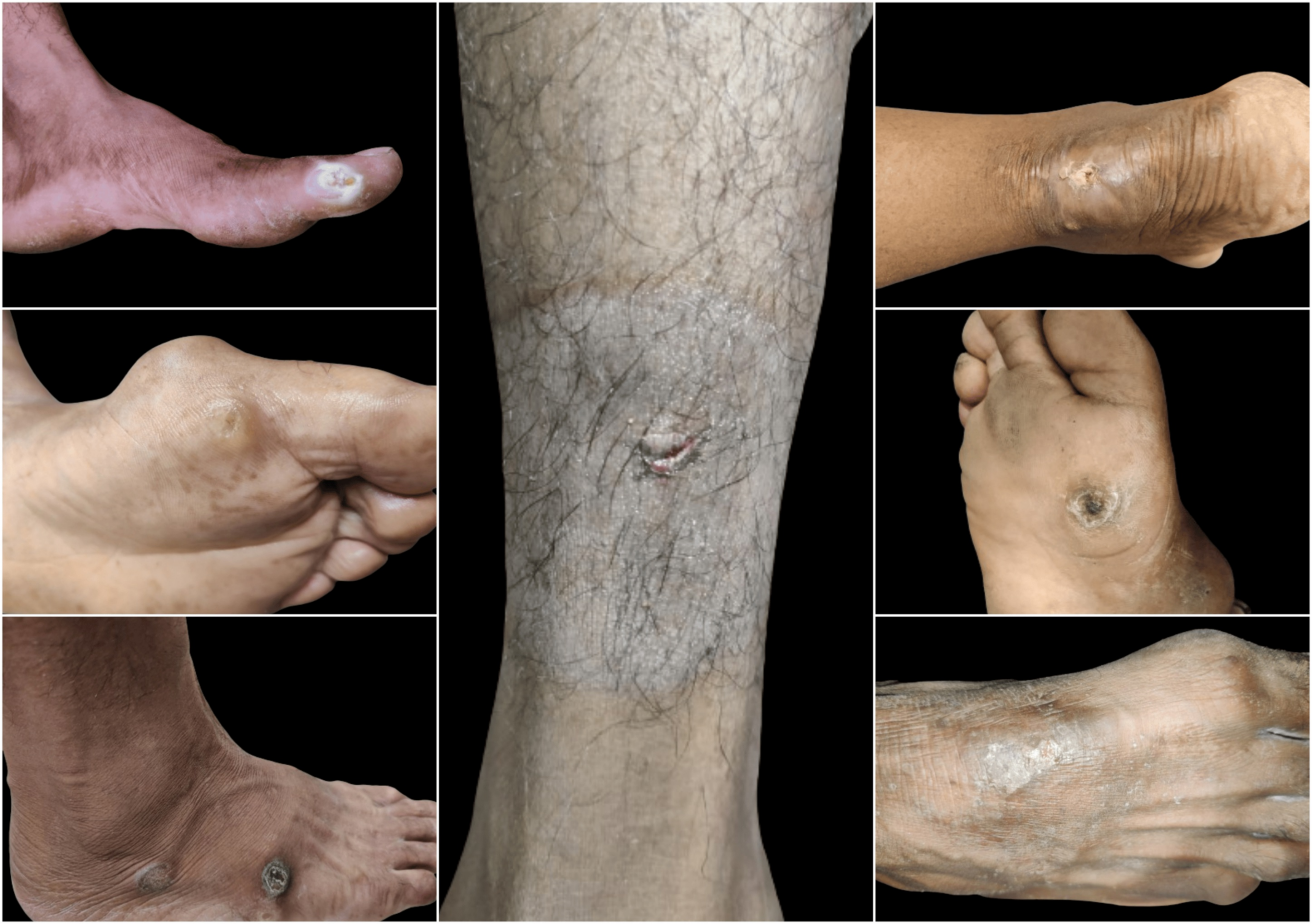
The patients with uncared traumatic wounds found in the present community-based study.

**Figure 5:**
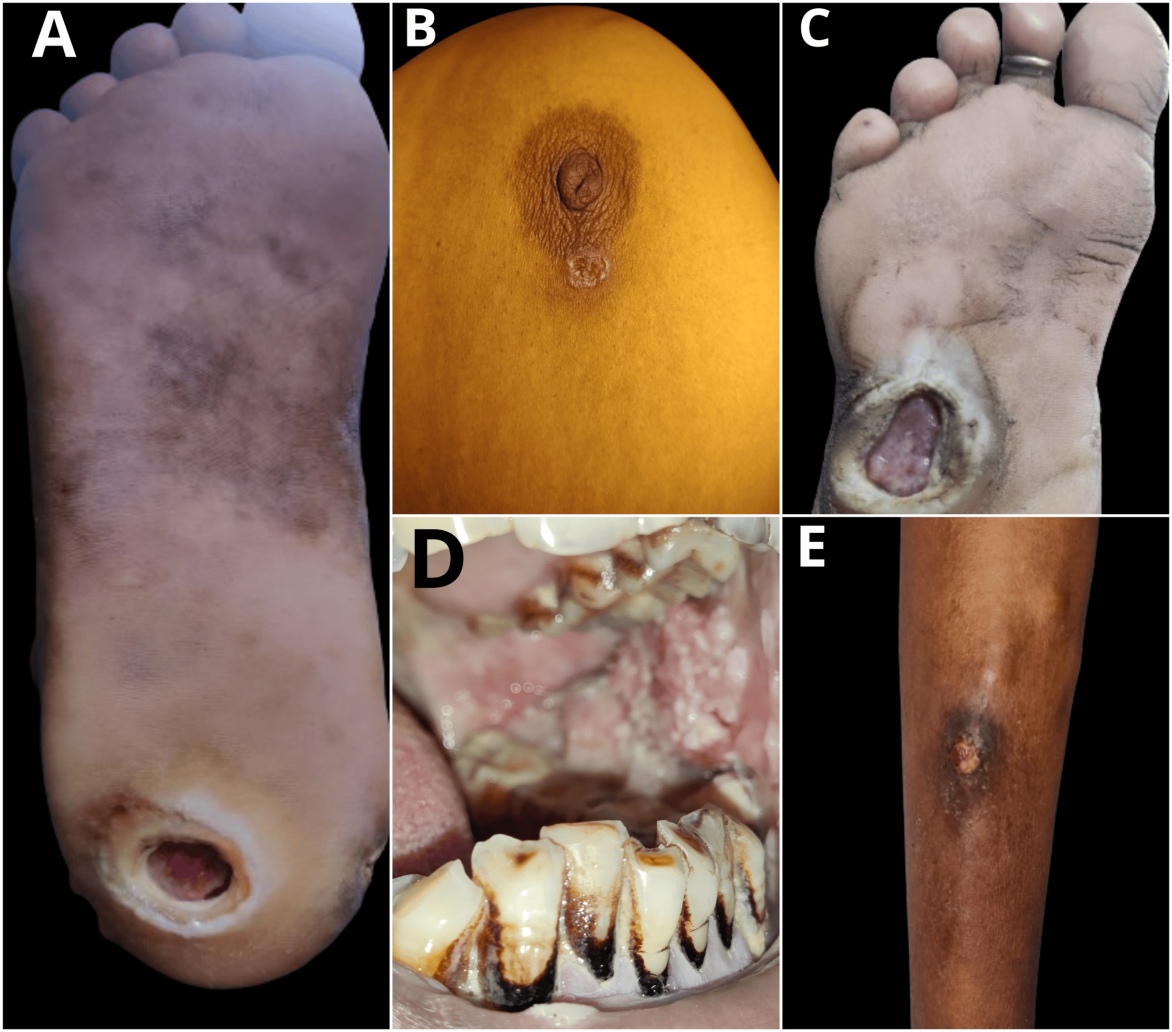
Other types of wounds found in the present community-based study. (A) A patient with a trophic ulcer in the right foot; (B) a tubercular ulcer over the right breast; (C) a neuropathic ulcer in the right foot; (D) a patient with carcinoma oral cavity; (E) uncared cellulitis in the right lower limb.

In the majority of the patients with chronic non-healing wounds, i.e., 10 (52.7%), the dimensions of the wounds were more than 5 cm (p=0.80). In around 16 (84.2%), the edge of the wound was sloping, and the floor of the wound was unhealthy and slowly healing in 15 (78.9%) of the patients (p=0.03). It was associated with tenderness in 15 (78.9%) of the patients (p=0.03), induration with no relation to deeper structures around the wound in 18 (94.7%) (p=0.004), and no associated bleed in 17 (89.5%) of the patients (p=0.007). Out of the total 19 patients having wounds, lymphadenopathy was present only in three (15.8%), of which two (10.5%) had palpable cervical nodes that were discrete, while one (5.3%) had palpable inguinal nodes that were matted. The patients with chronic non-healing wounds also had significant skin changes around the wounds, with seven (36.8%) patients having normal skin around the wounds. In the majority of the patients, i.e., seven (36.8%), the skin around the wound was tense and shiny; in three (15.8%) patients, the surrounding skin had pre-gangrenous changes; one (5.3%) patient had hair loss in the surrounding region of the wounds; and only one (5.3%) patients had developed gangrenous changes in the skin surrounding the wounds. The various types of wounds encountered during the community-based surveys in the urban (Sunderpur) and rural (Kandwa) communities of Varanasi district, Uttar Pradesh, India, are shown in Figures 3-5.

The overall characteristics of wounds in the study population have been shown in detail in Table 3.

**Table 3:** Wound characteristics in the patients with chronic non-healing wounds. p-value <0.05 is significant. *No observation DFUs, diabetic foot ulcers

Characteristics of wounds	June 2022-December 2023		p-value
	Frequency (N)	Percentage (%)	
Onset of wounds			
Spontaneous	16	84.2	0.0142
Trauma	3	15.8	
Total	19	100.0	
Location of wounds			
Other location	02	10.6	0.0065
Lower extremity	17	89.4	
Etiology associated with wounds			
Repeated trauma/uncared traumatic	7	36.8	
DFU (uncared)	7	36.8	
Leprosy	*	*	
Tubercular	1	5.3	
Post-cellulitis wound (uncared)	1	5.3	-
Trophic ulcer	1	5.3	
Arterial ulcer	*	*	
Venous ulcer	1	5.3	
Malignancy	1	5.3	
Total	19	100.0	
Duration of wounds			
3 to 6 months	14	73.68	0.06
More than 6 months	5	26.32	
Pain associated with wounds			
Absent	1	5.3	0.003
Present	18	94.7	
Discharge in wounds			
Present	5	26.3	0.06
Absent	14	73.7	
Type of discharge in wounds			
Absent	14	73.7	
Serous	*	*	
Hemorrhagic	*	*	-
Pus	5	26.3	
Total	19	100.0	
Personal history			
Smoker	7	36.9	0.27
Non-smoker	12	63.1	
Total	19	100.0	
Alcoholic	6	31.5	0.13
Non-alcoholic	13	68.5	
Total	19	100.0	
Vegetarian	8	42.1	0.49
Non-vegetarian	11	57.9	
Total	19	100.0	
Place of treatment			
Private practitioner	5	26.3	0.10
Public sector	13	68.4	
Type of treatment			
Local	2	10.6	
Systemic	*	*	
Local + systemic	16	84.2	-
Operative treatment	1	5.2	
Total	19	100.0	
Dimensions of wounds			
<5 cm	9	47.3	0.80
More than 5 cm	10	52.7	
Edge of wound			
Undermined	2	10.5	
Punched out	*	*	
Sloping	16	84.2	-
Raised pearly white beaded edge	1	5.3	
Everted	*	*	
Total	19	100.0	
Floor of wound			
Healthy granulating/debris	4	21.1	0.03
Unhealthy large slowly healing	15	78.9	
Tenderness			
Present	15	78.9	0.03
Absent	4	21.1	
Base			
Induration	18	94.7	0.004
No induration	1	5.3	
Relation with deeper structures			
Yes	1	5.3	0.004
No	18	94.7	
Bleeding (on touch)			
Yes	2	10.5	0.007
No	17	89.5	
Group of lymph nodes			
Inguinal nodes (matted)	1	5.3	
Cervical nodes (discrete)	2	10.5	-
Non-significant nodes	16	84.2	
Total	19	100.0	
Character			
Local temperature	*	*	
Shininess	7	36.8	
Loss of hair	1	5.3	-
Pregangrene	3	15.8	
Gangrene	1	5.3	
Normal	7	36.8	
Total	19	100.0	

General health based on the SF-36 questionnaire

In the present study, the majority of the patients, i.e., 11 (57.9%), reported their general health status as fair after developing chronic non-healing wounds, followed by five (26.3%) reporting their general health status as poor. In the majority (11, 57.89%) of the patients, the SF-36 questionnaire score was in the 40-50 range, while in five (26.3%) of 19 patients, it was in the 30-40 (poor) range regarding the QoL. The overall general health status of the patients with chronic non-healing wounds is shown in Table 4.

**Table 4:** Overall general health status of the patients with chronic non-healing wounds. *No observation

General health	Health of the patients at present compared with one year ago		
	SF-36 score (max score: 100)	Frequency	Percentage
Excellent	>80	*	*
Very good	70-80	*	*
Good	50-60	3	15.8
Fair	40-50	11	57.9
Poor	30-40	5	26.3
Total		19	100.0

In the present study, the majority of the patients, i.e., 13 (68.4%), reported little limitation in carrying out day-to-day activities, and nine (47.4%) patients with chronic non-healing wounds reported non-engagement in other recreational activities as they used to do before developing chronic wounds, while 10 (52.6%) reported no limitations in such engagements. Nearly 10 (52.6%) patients with chronic non-healing wounds reported a cutdown in social activities or as they used to do before developing chronic wounds. Only one (5.3%) patient reported no such dilemmas hindering their social domain of health. 

## Discussion

In the present study, only 19 patients were found to have chronic non-healing wounds, while acute wounds were not observed in any of the 10,003 individuals screened both in urban and rural communities. In our study, the overall prevalence of chronic wounds was 1.89 per 1000 (19/10003) people. The community-based study conducted in 2003, where 6917 individuals were screened, revealed that 104 patients had wounds, 73 had acute wounds, and 31 had chronic wounds [7]. Even though the previous study gave a clear demarcation about the prevalence of acute and chronic wounds, it could not draw a demarcation in order to give the prevalence of wounds in urban and rural settings separately [7]. In our study, we calculated the prevalence of wounds in urban areas, which was 1.57 per 1000 (11/6984) population, while in rural communities, it was 2.64 per 1000 (8/3019) population. The prevalence of wounds was higher in rural settings because of engagement in outdoor activities and poor health access, and most of them had neglected or uncared wounds [8,9].

In the majority of patients with wounds, i.e., 17 (89.4%), the most common anatomical location was the lower extremity, followed by other locations like the head and neck body and trunk regions, which constituted one (5.3%) affected patient each (p=0.0065). The majority, seven (36.8%) each, had uncared DFUs and neglected/uncared repeated trauma as the etiology for chronic wounds. The remaining five (26.31%) patients were equally divided between cellulitis, venous ulcers, trophic ulcers, tubercular ulcers, and malignancy, one (5.3%) each, as the etiology for chronic wounds. These findings were consistent with the study conducted by Gupta et al., including other studies where diabetics outnumbered all other causes of chronic wounds, respectively [7,10-12]. In our study, we did not encounter any acute wounds (<3 months duration) in any of the age groups, both in urban and rural communities. This may be attributed to the fact that prompt attention and treatment were received by such patients in various healthcare setups, private or public. The majority of the patients, i.e., 13 (68.4%), received primary treatment for wounds from the public sector, followed by five (26.3%) patients who received treatment from private practitioners, and only one (5.3%) adopted home remedies for the treatment of wounds. This is in contrast to the studies conducted previously, where 58.68% of the total patients with wounds opted for treatment based on home remedies [7].

The switch from opting for home-based remedies to supervised care under the clinician (be it in a private or public setting) clearly indicates that with time, people have shown interest in healthcare facility treatment under supervision over adopting home remedies for wound treatment, despite the fact that costs associated with the latter were on the higher side. The same domain may be attributed to the prompt management and efficacy of the management protocols by the treating physicians [10,11]. It is striking to see that in the present study, none of the patients in the screened population had wounds associated with arterial etiology; only one (5.3%) patient had venous ulcers present in the screened population. Similar findings were observed in the study conducted in 2003, where only 3.23% of the patients had arterial ulcers and none had venous ulcers out of the screened population [7].

In the present study, we estimated the QoL in patients with chronic non-healing wounds. The majority of the patients, i.e., 11 (57.9%), reported their general health status as fair after developing chronic non-healing wounds, followed by five (26.3%) reporting their general health status as poor. Only three (15.8%) patients with wounds reported their general health condition as good. In the majority, 11 (57.89%), the SF-36 questionnaire score was in the 40-50 range, while in the five (26.3%), it was in the 30-40 (poor) range. In only three (15.89%), the score was 50-60 in the good range regarding the QoL. These findings are in alignment with a study conducted by Shukla et al. in northern India [10]. The finding is also supported by a study on chronic wound patients from Essen University Hospital (the certified wound outpatient departments of the Department of Dermatology, Venerology and Allergology, the Vascular Medicine Department of the West German Heart Centre, and the Division of Vascular and Endovascular Surgery of the General, Visceral and Transplantation Surgery Department) and one in Dortmund (Foot Centre of the municipal clinics), which found a significant negative correlation between the total wound QoL score and patients with arterial leg ulcers, which reported that age affected Health Related Quality of Life (HRQoL), particularly for physical functions, in patients with DFUs [13]. The males and the patients with higher educational status were found to have a higher QoL when compared with those at the bottom line. The comorbidities like type 2 diabetes mellitus significantly contributed to the lower QoL in the patients with chronic non-healing wounds. The findings of our study on QoL were supported by the study conducted by Al Ayed et al. in which the patients with DFU revealed an overall lower HRQoL relating to all eight aspects of the SF-36 questionnaire [14].

The present study can help academicians examine a wider range of data sets and approach the issues at hand from various angles. However, such studies can act as a precedent and can lead to more opportunities for research and the creation of comprehensive, multidisciplinary data sets that can serve as warning signs for policymakers in developing multidisciplinary wound healing mechanisms and improving public health awareness for the best possible outcomes.

Limitations of the study 

Despite successfully carrying out the present study on a large scale, i.e., a screened population of 10,003 individuals for the prevalence of wounds over a 1.5-year period, we submit the potential limitations of the present study. In the present study, we used the census data available for the year 2011, as no authentic or government-published census data after 2011 was available for carrying out a community-based study. The rural population of the area, Kandwa village, has over a period of time escalated from a suburban to an urban level in Varanasi District. Hence, the rural extrapolation of the data may not be true.

However, we chose the above-mentioned areas as there is a university healthcare center in Sunderpur (urban) and Kandwa (rural), and the same regions were studied in the study conducted by Gupta et al. in 2003 [7]. As there was no study reported from other places/districts of India, we cannot generalize the data to the Indian scenario. But, the methodology of this study can be replicated in other parts of the country to have more robust pan-Indian data.

## Conclusions

The findings of the present study regarding the incidence and prevalence of chronic wounds in connection to demographic factors are important when allocating resources and making healthcare plans. The overall prevalence of wounds has decreased from 15.03 to 1.89 per 1000 people in India. The switch from opting for home-based remedies (turmeric powder, honey, neem oil, and others) to supervised care under the clinician (be it in a private or public setting) clearly indicates raised awareness regarding the management of wounds, and with time, people in the study area have shown an increased interest in healthcare facility treatment under supervision over adopting home remedies for wound treatment. The findings of the present study regarding the incidence and prevalence of chronic wounds in connection with demographic factors are important when allocating resources and making healthcare plans. The present study conveys a clear message to healthcare practitioners about the necessity of carrying out large-scale epidemiological research on this topic and offers a solid foundation for future studies. 

## Appendices

**Table 5:** (A) Proforma A for socio-demographic characteristics; (B) Proforma B for individuals having wounds.

	PROFORMA - A																								
	(FOR SOCIO-DEMOGRAPHIC CHARACTERISTICS)																								
	House Number:																								
	Head of the Family:																								
	Main Occupation of the Family:																								
	Total Income of the Family:																								
	Are members of whole family/Head of Family living in this same area for the past 20 years:																								
	Any member of family suffering from any Chronic Illness or is in Immunocompromised State:																								
	Community:																								
	Family Profile:																								
S.No.			Name of the Family Member		Age		Sex	Marital Status			Occupation		Addiction (If Any)			Literacy Status				Wound (Yes/No)			Wound Type		
PROFORMA - B																									
(FOR INDIVIDUALS HAVING WOUNDS)																									
		Type of wound:				Acute (<6 weeks)				Chronic (>6 weeks)															
		History:																							
		(A) Mode of Onset				A1 Spontaneous				A2 Trauma															
		(B) Duration				………… Years ………… Months ………… Days																			
		(C) Pain				Pain Score																			
						C1 Absent 0																			
						C2 Mild 1																			
						C3 Moderate 2																			
						C4 Severe 3																			
		(D) Discharge (Yes or No)										Type of Discharge (DT)													
		D1 Present										DT1 Serous													
		D2 Absent										DT2 Hemorrhagic													
		D3 Amount										DT3 Pus													
		Associated Disease (Yes or No)																							
		E1 Tuberculosis		E2 Diabetes					E3 Hypertension			E4 Leprosy			E5 Malignancy						E6 Peripheral Ischemic Disease				
		(F) Personal History										(G) History of treatment													
		F1 Smoker										G1 Type of Therapy:													
		F2 Non-Smoker										G1a Local			G1b Systemic						G1c Local + Systemic				
		F3 Alcoholic										G2 Duration													
		F4 Non-Alcoholic										G3 Treatment Cost													
		F6 Non-Vegetarian										G4 Health Provider													
												G4a Public Sector			G4b Private Practitioner						G4c Home Remedy				
		(H) Examination										(I) Pulse													
		H1 Pulse					H2 Blood Pressure					I1 Femoral			I3 Posterior Tibial						I2 Popliteal				
		H3 Respiratory Rate					H4 Temperature					I4 Anterior Tibial		I5 Dorsalis Pedis			I6 All Absent					I7 All Present			
		(J) General Examination																							
		J1 Pallor					J2 Icterus					J3 Cyanosis							J4 Clubbing						
		J5 Dehydrated							J6 Pedal Edema						J7 Lymphadenopathy										
		(K) Local Examination																							
		(K1) Anatomical location of wound										(K2) Wound Size (cm^2^)													
		K1a Head and neck										K2a <5 cm													
		K1b Body and trunk										K2b 5-10 cm													
		K1c Upper Extremity										K2c >10 cm													
		K1d Lower Extremity																							
		(K3) Edge										(K4) Floor													
		K3a Undermined										K4a Healthy granulating wound													
		K3b Punched Out										K4b Unhealthy large slowly healing wound													
		K3c Sloping										K4C Debris													
		K3d Raised pearly white beaded edge																							
		K3e Everted																							
		(K5) Discharges										(K6) Surrounding areas													
		K5a Amount										K6a Glossy red & edematous													
		K5b Colour										K6b Eczematous													
		K5c Odour										K6c Scar & wrinkling													
												K6d Induration													
												K6e Callus													
												K6f Normal													
		(K7) Tenderness										(K8) Base													
		K7a Present					K7b Absent					K8a Induration							K8b No Induration						
		(K9) Relation with deeper structures										(K10) Bleeding (on touch)													
												K10a Yes							K10b No						
		(K11) Associated Structure/s										(K12) Examination of Lymph Nodes													
												K12a Region			K12b Number						K12c Matted				
												K12d Tenderness			K12e Consistency						K12f Mobility				
		(L) Skin Changes										(M) Examination for Nerve Lesion													
		L1 Temperature					L2 Shininess					M1 Sensory													
		L3 Loss of hair					L4 Pregangrene					M2 Motor													
		L5 Gangrene					L6 Normal																		
		(N) Any other findings or comments																							
		INVESTIGATIONS																							
		Hemoglobin (g%)										Total Protein													
		Total Leucocyte Count										Albumin/Globulin													
		Differential Leucocyte Count										Blood Sugar													
		ESR										Blood Urea													
		BIOPSY & MICROBIOLOGY										RADIOLOGICAL INVESTIGATIONS													
		Histology					Swab Culture from the wound					Involved Part						Chest X-Ray (PA View)							

**Table 6:** Proforma C for assessment of QoL of individuals having wounds using the SF-36 questionnaire. QoL, quality of life

PROFORMA – C												
(FOR INDIVIDUALS HAVING WOUNDS; SF-36 QUESTIONNAIRE)												
Name:			Age:				Gender:			Type of Wound:		
Please answer the 36 questions of the Health Survey completely, honestly, and without interruptions.												
GENERAL HEALTH												
In general, would you say your health is:												
Excellent		Very Good				Good		Fair			Poor	
Compared to one year ago, how would you rate your health in general now?												
Much better now than one year ago							Somewhat better now than one year ago					
About the same							Somewhat worse now than one year ago					
Much worse than one year ago												
LIMITATIONS OF ACTIVITIES												
The following items are about activities you might do during a typical day. Does your health now limit you in these activities? If so, how much?												
Vigorous activities, such as running, lifting heavy objects, participating in strenuous sports.												
Yes, Limited a lot					Yes, Limited a Little				No, Not Limited at all			
Moderate activities, such as moving a table, pushing a vacuum cleaner, bowling, or playing golf												
Yes, Limited a lot					Yes, Limited a Little				No, Not Limited at all			
Lifting or carrying groceries												
Yes, Limited a lot					Yes, Limited a Little				No, Not Limited at all			
Climbing several flights of stairs												
Yes, Limited a lot					Yes, Limited a Little				No, Not Limited at all			
Climbing one flight of stairs												
Yes, Limited a lot					Yes, Limited a Little				No, Not Limited at all			
Bending, kneeling, or stooping												
Yes, Limited a lot					Yes, Limited a Little				No, Not Limited at all			
Walking more than a mile												
Yes, Limited a lot					Yes, Limited a Little				No, Not Limited at all			
Walking several blocks												
Yes, Limited a lot					Yes, Limited a Little				No, Not Limited at all			
Walking one block												
Yes, Limited a lot					Yes, Limited a Little				No, Not Limited at all			
Bathing or dressing yourself												
Yes, Limited a lot				Yes, Limited a Little					No, Not Limited at all			
PHYSICAL HEALTH PROBLEMS												
During the past 4 weeks, have you had any of the following problems with your work or other regular daily activities as a result of your physical health?												
Cut down the amount of time you spend on work or other activities												
Yes							No					
Accomplished less than you would like												
Yes							No					
Were limited in the kind of work or other activities												
Yes							No					
Had difficulty performing the work or other activities (for example, it took extra effort)												
Yes							No					
EMOTIONAL HEALTH PROBLEMS												
During the past 4 weeks, have you had any of the following problems with your work or other regular daily activities as a result of any emotional problems (such as feeling depressed or anxious)?												
Cut down the amount of time you spend on work or other activities												
Yes							No					
Accomplished less than you would like												
Yes							No					
Didn't do work or other activities as carefully as usual												
Yes							No					
SOCIAL ACTIVITIES												
Emotional problems interfered with your normal social activities with family, friends, neighbors, or groups?												
Not at all		Slightly				Moderately		Severe			Very Severe	
PAIN												
How much bodily pain have you had during the past 4 weeks?												
None	Very Mild				Mild		Moderate		Severe			Very Severe
During the past 4 weeks, how much did pain interfere with your normal work (including both work outside the home and housework)?												
Not at all		A little bit				Moderately		Quite a bit			Extremely	
ENERGY AND EMOTIONS												
These questions are about how you feel and how things have been with you during the last 4 weeks. For each question, please give the answer that comes closest to the way you have been feeling.												
Did you feel full of pep?												
All of the time	Most of the time			A good Bit of the Time			Some of the time		A little bit of the time			None of the Time
Have you been a very nervous person?												
All of the time	Most of the time			A good Bit of the Time			Some of the time		A little bit of the time			None of the Time
Have you felt so down in the dumps that nothing could cheer you up?												
All of the time	Most of the time			A good Bit of the Time			Some of the time		A little bit of the time			None of the Time
Have you felt calm and peaceful?												
All of the time	Most of the time			A good Bit of the Time			Some of the time		A little bit of the time			None of the Time
Did you have a lot of energy?												
All of the time	Most of the time			A good Bit of the Time			Some of the time		A little bit of the time			None of the Time
Have you felt downhearted and blue?												
All of the time	Most of the time			A good Bit of the Time			Some of the time		A little bit of the time			None of the Time
Did you feel worn out?												
All of the time	Most of the time			A good Bit of the Time			Some of the time		A little bit of the time			None of the Time
Have you been a happy person?												
All of the time	Most of the time			A good Bit of the Time			Some of the time		A little bit of the time			None of the Time
Did you feel tired?												
All of the time	Most of the time			A good Bit of the Time			Some of the time		A little bit of the time			None of the Time
SOCIAL ACTIVITIES												
During the past 4 weeks, how much of the time have your physical health or emotional problems interfered with your social activities (like visiting with friends, relatives, etc.)?												
All of the time	Most of the time			A good Bit of the Time			Some of the time		A little bit of the time			None of the Time
GENERAL HEALTH												
How true or false is each of the following statements for you?												
I seem to get sick a little easier than other people												
Definitely true		Mostly true				Don't know		Mostly false			Definitely false	
I am as healthy as anybody I know												
Definitely true		Mostly true				Don't know		Mostly false			Definitely false	
I expect my health to get worse												
Definitely true		Mostly true				Don't know		Mostly false			Definitely false	
My health is excellent												
Definitely true		Mostly true				Don't know		Mostly false			Definitely false	
